# Impact of Fortified Malt-Based Food on Immunity Outcomes in School Children in India: Cluster Randomized Controlled Trial

**DOI:** 10.2196/54189

**Published:** 2025-06-25

**Authors:** Anuradha Khadilkar, Vinay Rawat, Jaladhi Bhatt, Devyani Chaturvedi, Vivek Garg, Pankaj Verma

**Affiliations:** 1 Jehangir Clinical Development Centre, Jehangir Hospital Premises, Bund Garden Road, Sangamvadi, Pune, India, 91 9850244305; 2 GlaxoSmithKline Consumer Healthcare Limited, Gurgaon, India; 3 Hindustan Unilever Limited, Gurgaon, India; 4 Unilever USA, Newark, CA, United States

**Keywords:** multinutrient supplement, study design, Indian children, immunity, fortified malt-based food, school-based

## Abstract

**Background:**

Nutritional inadequacy and consequent diminished immunity among school-age children is a public health problem in India. Nutrition interventional studies using a cluster randomized controlled trial (RCT) design can avoid ethical issues inherent in double-blind individual RCTs in children involving daily administration of an empty-calorie placebo.

**Objective:**

We tested the hypothesis that daily administration of a fortified malt-based food (FMBF), a multinutrient supplement, would improve immunity outcomes against common infectious diseases, nutritional status, and gut health in Indian school-age children by using a cluster RCT design. This report presents the study design attributes and the baseline characteristics of the study population.

**Methods:**

This was an open-label, 2-arm, parallel-group, matched-pair cluster RCT, stratified by gender, in children aged ≥7 to ≤10 years old with height-for-age *z* scores (HAZ) of ≥−3 to ≤−1 and good general health. Four schools located in Pune city in India participated in the study. Each school was deemed as a cluster and was randomized to the test group (FMBF and dietary counseling) or control group (dietary counseling alone). A total of 924 participants from the 4 randomized schools were enrolled in the study.

**Results:**

Observed mean age (SD) was 8.0 (SD 0.81; range: 7-10) years. There was no significant difference in mean age (*P*=.06), gender (*P*=.55), race (*P*>.99), HAZ category (*P*=.051), HAZ (*P*=.17), and BMI (*P*=.03). A very large proportion of children had micronutrient inadequacies in terms of vitamin D (97.5%), folate (79.2%), zinc (66%), and vitamin A (34.3%) at baseline. The study design meant that administration of the study intervention at a cluster level was easy. Mean compliance with the test product was 99.99% and retention in the study was 98%.

**Conclusions:**

The findings highlight the extent of nutritional inadequacies in Indian school-age children, reaffirming the need for nutritional strategies to optimize the nutritional status among these children. A cluster RCT design can be effectively used in nutritional intervention trials with children by maintaining high compliance and retention.

## Introduction

Nutritional inadequacy has been the major and long-standing cause of nutritional and health concerns and developmental shortcomings in Indian children [1]. Despite numerous health care advances, the burden of major communicable diseases in children continues to be high in India compared to other countries [2,3]. As per the 2021 Global Burden of Disease study, diarrheal diseases and respiratory infections among children (5‐14 y) were 2 of the 5 leading infectious causes of disability-adjusted life-years in the country [3]. Furthermore, child and maternal malnutrition (nutritional deficiencies, diarrhea, lower respiratory infections, and other common infectious diseases) were among the top 5 leading risk factors for disability-adjusted life-years [3].

In terms of nutritional status, approximately 60% of the Indian pediatric population has protein-energy malnutrition [4-8]. A recent nationwide survey further reported a deficiency in zinc, iron, and folate, as well as vitamins A, B12, and D in 14%‐32% of preschool children (aged 1-4 y), 17%‐28% of school-age children (aged 5‐9 y), and 16%‐37% of adolescents (aged 10‐19 y) [9].

Nutritional inadequacy hampers the overall development of children as it adversely affects their immunity, normal physiological functions, physical growth, and cognitive development [10-14]. Such inadequacies often coexist with infectious diseases and exhibit complex interactions, leading to a vicious cycle of malnutrition and infections [15-18]. This is evidenced by the fact that child undernutrition is responsible for 22% of the country’s burden of disease [19].

As per the recent Global Nutrition Report, India performs poorly across standard child nutritional measures, accounting for nearly 39% of stunting and 15% of wasting worldwide [20]. On the national level for children under 5 years of age, data from the 2015‐2016 National Family Health Survey showed a reduced prevalence of child stunting and underweight (38% and 36%) since 1992‐1993 (52% and 53%) [21], however, it is still alarmingly high considering the multisectoral nutritional policies and initiatives that have been implemented [22]. Although there is sufficient anthropometry-related nutritional data for the under-5 age group, there is a dearth of national-level anthropometric data for school-age children. The Comprehensive National Nutrition Survey (CNNS) was the first national survey to measure anthropometry in children aged 5 to 14 years. According to the CNNS 2016‐2019, the prevalence of stunting and underweight for children in the age group of 5‐9 years was nearly 22% and 35%, respectively. However, the prevalence of stunting and underweight varied with school status, with a higher percentage of both among children who were out of school compared to school-going children (stunting: 38% vs 20%; underweight: 45% vs 34%) [9].

Evidently, at the individual level, appropriate nutrition actions in children can help bridge the nutritional gap, reduce the morbidity burden through improved immunity, and contribute to their overall health during the crucial formative and learning years [13,23-31]. Nutritional supplementation is one of the widely accepted approaches out of several effective nutritional strategies available worldwide [32,33]. Of note, in the Indian scenario, nutritional supplements have mainly been studied for their effect on the nutritional status and immunity of children <5 years of age. However, since school-age children represent about one-fifth of the total Indian population [34], understanding the effect of nutritional supplements in this cohort is equally essential. In addition, of the existing literature, none of the studies have comprehensively evaluated any nutritional supplement for its overall effect on the growth and immunity/health outcomes of Indian school-age children [35-38].

Thus, we conducted a study to provide a comprehensive overview of one such supplement, a multinutrient fortified malt-based food (FMBF), for its effect on immunity as well as other health aspects, in combination with dietary counseling, in a large group (>900) of Indian school-age children (>7 and <10 y). This paper gives an overview of the study design and baseline characteristics of the study population.

## Methods

### Study Design

This was a single-center, multiple-site, open-label, parallel-group, school-based, matched-pair cluster randomized controlled trial (RCT) conducted with Indian school-age children aged ≥7 to ≤10 years (ClinicalTrials.gov registration number NCT02542865). The study was conducted over 10 visits that consisted of screening (visit 1), baseline assessment (visit 2), dietary counseling (visit 3 to 9), and an end-of-study visit (visit 10). Nutritional intervention with an FMBF for the test group was done every day from visit 3 to 9 over a period of 9 months.

After obtaining written permission from management boards, 4 nongovernment schools from Pune, India, with a coeducation system and no history of outbreaks/clusters of infectious disease cases in the prior month consented to participate in the study. Schools were matched for size (with respect to the number of children aged 7 to 10 y), socioeconomic profile of the students, and type of school (government-subsidized or private) and 2 matched pairs were formed. Each school was deemed a cluster and assigned a randomization number in ascending numerical order. A randomization schedule with details on treatment assignment was prepared by an independent statistician from the Biostatistics Department, GlaxoSmithKline Consumer Healthcare, using validated internal software. The researchers involved in the study did not know the details of the schedule. The schedule was stored and concealed in an internal electronic document management system. Based on the randomization schedule and school list, each school within the matched pairs was randomized to receive either the multinutrient FMBF along with dietary counseling (test group) or dietary counseling alone (control group) during the intervention phase. The study assessed the effect of the FMBF on the overall development of these school-age children (immunity, nutritional status, gut health, anthropometry, dietary status), along with the FMBF’s safety profile.

For quality control, all equipment and instruments were regularly calibrated, an accredited lab was used, and tools were validated. Staff were trained and retrained at regular intervals. To maintain the quality standards of data collection as per protocol and regulatory standards, data monitoring and data collection and analysis were done by a contract research organization. In addition, the study was audited by the Clinical Data Quality Assurance team at GlaxoSmithKline US and received an audit certificate.

### Ethical Considerations

The study was conducted in accordance with the International Council on Harmonization Guidelines for Good Clinical Practice and the Declaration of Helsinki. The protocol and all the amendments were approved by Ethics Committee Jehangir Clinical Development Centre Pvt Ltd (ECR/352/Inst/MW2013) prior to study initiation. Written informed consent was obtained from one of the parents or a legally acceptable representative (LAR) of each participant and written assent was obtained from all participants before any study-specific procedures were performed. Participants didnot receive any compensation for participating in the clinical study but all participants travel cost for study visit was reimbursed as per approved amount by Ethics Committee and same was mentioned in Informed Consent. The data collected for clinical study was stored by protecting privacy and maintaining confidentiality.

### Study Inclusion and Exclusion Criteria

The key study inclusion and exclusion criteria are summarized in Textbox 1.

Textbox 1. **Key study eligibility criteria.**

**Inclusion criteria**

- Children of either gender
- Children aged 7 to 10 years (inclusive) with height-for-age *z* scores (HAZ) between −3 and -1 (inclusive)
- Children with good general and mental health in the opinion of the investigator or a medically qualified designee, defined as: (1) no clinically significant/relevant abnormalities in medical history or upon physical examination and (2) absence of any condition that could affect the child’s safety/well-being or their ability to understand the study aspects

**Exclusion criteria**

- Children in care
- Children with an allergy or intolerance to any ingredient of the fortified malt-based food
- Children with severe anemia (hemoglobin <8 g/dL)
- Children with a history of use of immunosuppressive therapy (eg, oral corticosteroids or chemotherapy) in the 6 months prior to the screening visit
- Children with a recent history (2 months) of serious infections, injuries, and/or surgeries
- Children with a current or relevant history of any serious, severe, or unstable physical or psychiatric illness or any medical disorder that could have made them unlikely to complete the study
- Children who have consumed nutritional supplements and/or health food drinks on a regular basis (≥3 times a week) in the last 3 months
- Children with any other condition that presented an undue risk to them from the study interventions or procedures, in the opinion of the investigator or medically qualified designee

### Study Interventions

The FMBF was prepackaged in sachets, each containing 27 g of multinutrient beverage powder. The nutrition profile of this supplement is presented in Table S1 in Multimedia Appendix 1. Participants from the test group consumed 27 g of this powder mixed in 150 mL of lukewarm water twice per day for 9 months. To avoid stomach fullness as a deterrent against compliance, the interval between the 2 doses was maximized. One dose was administered as soon as the participants entered the school and the second dose was given just prior to school dismissal. When a child was absent from school (eg, due to holidays or sickness), parents/LARS administered the 2 doses at home (one in the morning, one in the evening).

Nutritional inadequacy and the resulting immunity outcomes could be influenced by dietetic interventions implemented to improve nutritional intake. Background diet is a major confounder and it is imperative to track dietary quality throughout the study. Thus, to keep the background diets comparable between the 2 groups, the test group was administered the test product, while both the test and control group received dietary counseling. All study participants, their teachers, and their parents/LARs received age-specific dietary counseling to optimize the nutritional intake of the participants, which was based on the dietary guidelines for Indians established by the Indian Council of Medical Research–National Institute of Nutrition [39]. A total of 7 separate sessions of dietary counseling were administered to study participants and parents/LARs in both groups. The first 2 sessions involved the delivery of content and were mandatory to attend. The remaining 5 sessions were for follow-up, reinforcement, and problem-solving of content delivered in the first 2 sessions and these sessions were not mandatory to attend. The quality, content, and duration of the dietary counseling were equivalent in both the study groups.

### Study End Points

The primary efficacy end point assessed the total number of ill days due to gastrointestinal (GI) and respiratory illness episodes in the participants over 9 months. GI illness was defined as an acute illness that included any of following symptoms: 3 or more loose, liquid, or watery stools or any vomiting in 24 hours [40]. Diarrhea or vomiting arising from noninfectious causes, such as irritable bowel syndrome or medications, were excluded. Respiratory illness was defined as an acute illness that included ≥1 of the following symptoms: runny nose, stuffy or blocked nose, cough, fever or chills, sore throat, or sneezing [41]. Symptoms arising due to noninfectious causes (eg, allergy) were excluded from the diagnosis. The secondary efficacy end points evaluated over 9 months included assessment of frequency and severity of the GI and respiratory illness episodes, school absenteeism due to these episodes, change from baseline in serum micronutrient levels (vitamins A, B12, D, and E, folate, and trace elements zinc, iron, copper, and selenium), change from baseline in macronutrient status, change from baseline in dietary diversity assessed using 24-hour individual dietary diversity score (IDDS), change from baseline in gut integrity as measured by a lactulose:mannitol test and urinary neopterin test, and change from baseline in BMI. Details of the evaluation of secondary end points are presented in Table 1. Safety assessments included an evaluation of adverse events (AEs), treatment-emergent AEs, and serious AEs throughout the study. The laboratory assessments, vital signs, physical examinations, and hemoglobin measurements in these participants were conducted at baseline and at the end of the study.

**Table 1.** Secondary end point evaluation methods and tools.

Secondary efficacy end point	Frequency of assessment	Evaluation tool	Evaluation/test method
Frequency (number of episodes) of gastrointestinal and respiratory illnesses	Weekly basis during scheduled SPWR^a^ visits	Diagnosis form	Calculated as total number of gastrointestinal and respiratory illness episodes, divided by the duration of the intervention, where each episode is defined as each instance of illness followed by at least 3 symptom-free days [40,42,43].
Severity of an illness episode	Weekly basis during scheduled SPWR visits	Diagnosis form	Assessed as per severity grading of mild, moderate, and severe as outlined, evaluated, and classified by a study physician [44].
School absenteeism due to gastrointestinal and respiratory illnesses	Weekly basis during scheduled SPWR visits	Diagnosis form	Calculated as number of days when a child failed to attend school because of gastrointestinal and/or respiratory illnesses.
Change from baseline in BMI	Twice in the study (at screening and at the end of the study visit)	Portable stadiometer (SECA 213) for measuring a participant’s height. Standardized weighing scale (SECA 874) for measuring a participant’s weight.	Calculated using the formula BMI=weight [kg] ∕ (height[m])^2^. All anthropometric measurements were performed using the guidelines adopted at the National Institute of Health–sponsored Arlie Conference [45].
Change from baseline in gut integrity/health	Twice in the study (at baseline and at the end of the study visit)	Urine testing	Urine lactulose:mannitol assessment by HPLC^b^. Urinary neopterin assessment by ELISA^c^.
Change from baseline in mucosal immunity	Twice in the study (at baseline and at the end of the study visit)	Saliva testing	Salivary IgA^d^ assessment by ELISA
Change from baseline in levels of the micronutrients vitamins A, B12, D (25-hydroxycholecalciferol), and E, folate, and trace elements selenium, zinc, copper, and iron	Twice in the study (at baseline and at the end of the study visit)	Blood testing	Serum vitamin A, B12, D, and E, and folate assessment by ELISA Serum copper assessment by 3, 5-Dibromo-PAESA Serum zinc assessment by 5-Br-PAPS Serum iron assessment by TPTZ^e^ Serum ferritin assessment by turbidimetry Serum selenium assessment by ICP^f^
Change from baseline levels of ferritin, sTfR^g^, CRP^h^, and AGP^i^	Twice in the study (at baseline and at the end of the study visit)	Blood testing	Serum sTfR and CRP assessment by immunoturbidimetry Serum AGP assessment by turbidimetry
Change from baseline in dietary diversity score	Twice in the study (at baseline and at the end of the study visit)	24-hour dietary recall survey Dietary diversity questionnaire [46]	Based on data of the foods and beverages consumed in the last 24 hours as captured by a 24-hour dietary survey, the appropriate food groups in the questionnaire were selected. Dietary diversity scores were calculated by adding the number of food groups consumed by the child over the 24-hour recall period.
Change from baseline in energy, protein, carbohydrates, and fat consumption	Twice in the study (at baseline and at the end of the study visit)	24-hour dietary recall survey [47]	24-hour dietary recall survey

a SPWR: study physician weekly review.

b HPLC: high-performance liquid chromatography.

c ELISA: enzyme-linked immunosorbent assay.

d IgA: immunoglobulin A.

e TPTZ: 2,4,6-Tripyridyl-S-triazine.

f ICP: inductively coupled plasma.

g sTfR: soluble transferrin receptor.

h CRP: c-reactive protein.

i AGP: alpha-1-acid glycoprotein.

### End Point Assessments

Study end point assessments were performed as per the study schedule presented in Table 2.

**Table 2.** Study baseline and end point assessment schedule.^a^

Activity	Visit 1^b^	Visit 2^c^	Dietary counseling visits^d^ (visits 3-9)^e^	Visit 10^f^
Informed consent and assent^g^	✓			
Demographics	✓			
Medical history	✓			
Current/concomitant medication	✓	✓	✓	✓
General physical examination	✓			✓
Vital signs	✓			✓
Hemoglobin assessment using Pronto	✓			
Anthropometric measurements (height, weight, BMI, and height-for-age *z* scores)	✓			✓
Inclusion/exclusion criteria evaluation	✓			
Participant eligibility	✓			
Continued eligibility criteria		✓	✓	✓
24-hour dietary recall and dietary diversity survey	✓			✓
Parental/LAR training to complete “symptom checklist”^h^	✓			
Dispense blank parent/LAR symptom checklist and product compliance report forms^i^	✓			
Sample collection for urinary neopterin test and lactulose:mannitol test		✓		✓
Sample collection for analysis of serum ferritin, serum transferrin receptor, c-reactive protein, alpha-1-acid glycoprotein, salivary immunoglobulin A^j^, and nutritional biochemistry		✓		✓
Dietary counseling			✓	
Product administration (applicable to the test group only)^k^			✓	
Product compliance check and collecting empty product sachets (applicable to test group only)			✓	
Adverse event monitoring		✓	✓	✓
Study completion and medical sign off				✓

a Study Physician Weekly Review was initiated 1 week (+ up to 2 days) post first dietary counselling visit of the participant and was continued on a weekly basis (+ up to 2 days) until end of study visit.

b Visit 1: screening visit.

c Visit 2: baseline visit (+1 to 21 days post–screening visit).

d Sessions number 3, 4, 5, 6, and 7 could be postponed in view of long school holidays and exams. Session number 7 was not to be conducted any later than the last day of month 8 of the intervention period. A minimum of a 4-week gap was maintained between sessions number 2, 3, 4, 5, 6, and 7.

e Visit 3/session 1: day 1 to 3 of week 1 from baseline visit; visit 4/session 2: week 2 from baseline visit and up to 3 days; visit 5/session 3: week 6 from baseline visit and up to 3 days; visit 6/session 4: week 10 from baseline visit and up to 3 days; visit 7/session 5: week 14 from baseline visit and up to 3 days; visit 8/session 6: week 18 from baseline visit and up to 3 days; visit 9/session 7: week 22 from baseline visit and up to 3 days.

f Visit 10: end of study visit (+1 to 7 days after the end of 9 months from the baseline visit).

g Obtaining informed consent and assent and performing screening activities could be on different dates but informed consent and assent were obtained prior to performing and applying any screening criteria or procedures. Screening activities were done within 60 days of obtaining consent and assent.

h Could be done any time after obtaining consent and assent but prior to baseline visit.

i Parent/legally acceptable representative symptom checklist and product compliance report forms were collected and reviewed for each weekly review with the physician.

j Saliva samples were collected for the assessment of salivary immunoglobulin A at visit 2 and visit 10.

k Intervention began +1 to 3 days from the baseline visit and continued for 9 months. Compliance checks (test product empty sachets were collected) and AEs were recorded throughout the intervention period.

To evaluate primary and secondary immunity outcomes, the parents/LARs of the participants regularly updated the symptom checklist and compliance report forms for study interventions, which were collected and analyzed during each study physician weekly review (SPWR) throughout the study. During the SPWR, the start/end date of GI and respiratory illness episodes along with the frequency, severity (mild, moderate, severe), and school absenteeism due to these episodes were noted in the diagnosis form by the study physicians based on details obtained from the following:

- Symptom checklist completed by parents/LARs
- External clinical record if treated by an external physician
- Direct information from participants or parents/LARs
- Clinical history and examination by study physician

Details of the evaluation methods and tools used for assessment of secondary immunity outcome end points, anthropometry, serum biochemistry, and dietary assessment are provided in Table 1.

During the study, the AEs and serious AEs were monitored in addition to those reported by participants or parents/LARs to the study physicians as and when observed or through the weekly symptom checklists. The AEs were regarded as treatment-emergent AEs if they occurred on or after the first administration of the FMBF in the test group or on or after the first dietary counseling session in the control group.

A compliance check of the study interventions was conducted as per the schedule presented in Table 2. Concomitant medications were recorded at each study visit. Except for immunosuppressive therapies (which were never used in the study), there were no restrictions on medications and medical treatments. However, the use of medications (eg, antibiotics, antipyretics) was standardized across all study sites.

A noninvasive, spot-check monitoring device (Masimo Corporation’s Pronto Pulse CO-Oximeter) was used to screen study participants in school settings based on their hemoglobin (Hb). This device uses transcutaneous spectrophotometry to measure Hb concentration in the blood, and the result is comparable to invasive laboratory-based Hb estimations in children [48]. Anthropometric measurements included the assessment of height, weight, BMI, and HAZ. HAZ was obtained using the World Health Organization Anthroplus software [49].

### Sample Size

The sample size was based on the primary efficacy end point, the total number of ill days due to GI and respiratory illnesses. Based on the data from the Global Burden of Disease by the World Health Organization [50], the Institute for Health Metrics and Evaluation [51], and the Census of India [34], approximately 7 ill days due to GI and respiratory illnesses per 9 months was assumed in this study population. Thus, the sample size was calculated based on the assumption of an average of 7 ill days in the control group with a difference of 10% (0.7 d) between the study groups.

No sample size considerations were taken into account for secondary objectives. The simulated data were also used based upon the assumption of a Poisson distribution per study treatment. On observation of these simulated data, it was considered that these Poisson distributions were not heavily skewed and, as a result, normal distributions could be assumed. Consequently, a normality assumption was adopted for both the generation of sample size and the intended approach to the statistical analysis. Because of the assumption of Poisson distribution, estimates of variability were calculated as the square root of the mean. The mean of the control was assumed to be 7.0 (SD 2.65), while the mean of the test group was 6.3 (SD 2.51). In order to achieve 80% power, 215 participants per treatment arm (total=430) were required to complete the study in case of individual randomization. This assumed a 5% level of significance (2-tailed *t* test). As this study used cluster randomization, to retain equivalent power to an individually randomized trial, design effect (DE), which is a function of the intracluster correlation coefficient (ICC), was considered for sample size calculations. The scientific literature suggested that DE due to a cluster sampling strategy should be assumed to be 1.5 [52]. A similar assumption was made in a study that used 3-stage systematic cluster sampling [53]. The following formula was used [54]:


$${\text{N}}^{*}=\text{N}\times \text{DE}$$

Where N* is the number of participants required for a cluster RCT, N is the number of participants required for an individual RCT, and DE is the design effect.

Since DE was assumed to be 1.5, the number of evaluable participants required to complete the study was determined to be 323 per arm (total=646). To allow for a 30% dropout rate, a total of 924 participants were to be randomized. Approximately 1300 participants were to be screened to randomize approximately 924 participants in order to obtain 646 participants completing the study.

### Statistical Analysis

#### Analysis Population

Safety population was defined as participants who received ≥1 dose of the study interventions (only dietary counseling [control group] or both FMBF and dietary counseling [test group]) during the study. The intention-to-treat (ITT) population comprised all participants in the safety population with any postinterventional assessment (ie, SPWR), according to the study group assigned. The modified intention-to-treat (mITT) population was defined as all participants in the safety population with any postinterventional assessment (SPWR) who completed the entire intervention phase and attended the end-of-study visit.

#### Analysis Methods

Access to the treatment code was appropriately restricted during the process of blinded data review involving clinical research scientists and statisticians. The database with potentially unblinded information was separately reviewed for study population inclusion/exclusion by unblinded clinical research scientists. The database was later validated by a blinded reviewer prior to its release for analysis.

Analysis of the primary efficacy end point was done on the mITT population and the analysis of secondary and exploratory efficacy end points was done on both the mITT and ITT population. They were analyzed using the analysis of covariance (ANCOVA) model (SAS Studio version 9.4 or higher; SAS Institute Inc). The ANCOVA had cluster (school) as a random effect; study group and gender as fixed effects; and baseline assessments, baseline serum levels, and baseline IDDS as covariates. Unlike other study end points, frequencies for severity (mild, moderate, severe) of GI/respiratory illness episodes as well as IDDS categories (low, moderate, high) were compared using the *χ*^2^ test (if frequencies were >5%). If frequencies were ≤5% in any study group, then these were compared using the Fisher exact test. Adjusted means for each study group, 95% confidence intervals, within-group *P* values, difference between the study groups, 95% confidence intervals of the difference, and the between-group *P* values were also presented based on the above statistical model. All statistical tests of hypotheses were 2-sided and used a level of significance of α=.05.

Demographics, baseline characteristics, and safety assessment (including laboratory tests) summaries were produced for the safety, ITT, and mITT populations. Descriptive statistics including n (%), mean (SD), minimum, and maximum (as applicable) were provided. Baseline characteristics were compared between the treatment groups using an independent *t* test for continuous variables and *χ*^2^ test for categorical variables. A 2-sided significance level of 5% was used for the comparisons.

The intervention was targeted at the cluster level; however, outcomes were measured at the individual level. Thus, all ANCOVA analyses included cluster as a random effect to introduce correlation between children from the same school, which was estimated by the ICC. The assumptions of normality and homogeneity of variance were investigated. Violations of these assumptions were overcome using suitable transformation (eg, log).

## Results

### Overview

The study was conducted between July 2017 and July 2018. Recruitment of all participants was completed in a 4-month period. After screening and baseline assessment, each recruited participant remained in the study for 9 months to receive the interventions and undergo assessments.

The mean product compliance in the test group was 99.99% (SD 0.02, minimum 99.69% and maximum 100%). Mean compliance with both mandatory and follow-up sessions of dietary counseling for participants as well as parents/LARs was 100% in both the test and control groups.

### Demographic and Baseline Characteristics of Study Participants

**Figure 1.**
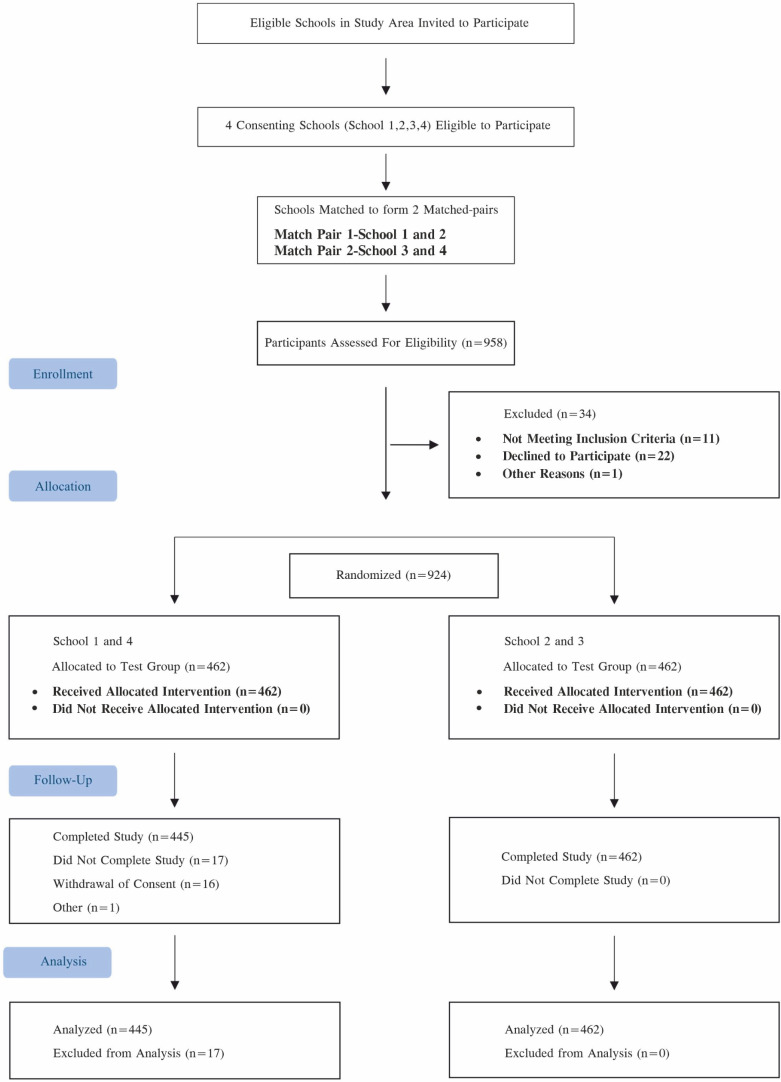
CONSORT flowchart of study design and participant disposition. CONSORT: Consolidated Standards of Reporting Trials.

Figure 1 depicts the study design and disposition of the study participants. Of 958 screened participants, 924 participants were randomized to the test group and the control group in a 1:1 proportion (n=462 each). A total of 907 participants (98.2%) completed the study (test: n=445; control: n=462), whereas 17 (3.7%) participants did not complete the study, either due to withdrawal of consent (test: n=16, 3.5%) or other unspecified reasons (test: n=1, 0.2%).

The demographic and baseline characteristics of the study participants are summarized in Table 3. All the participants had Central/South Asian heritage. Both the study groups (test vs control) were comparable in terms of mean age of the participants (8.0 y vs 7.9 y), gender distribution (male: 57.4% vs 55.4%), mean HAZ (−1.52 vs −1.48), and mean BMI (15.47 kg/m^2^ vs 15.12 kg/m^2^).

**Table 3.** Demographic and baseline characteristics of study participants (safety population).

Parameters	Test group (n=460)	Control group (n=462)	Total (n=922)	*P* value
Age, years				
Mean (SD)	8.0 (0.78)	7.9 (0.84)	8.0 (0.81)	.06
Range	7-10	7-9	7-10	
Gender, n (%)				
Female	196 (42.6)	206 (44.6)	402 (43.6)	.55
Male	264 (57.4)	256 (55.4)	520 (56.4)	
Race, n (%)				
Central Asian/South Asian heritage	460 (100)	462 (100)	922 (100)	>.99
HAZ^a^ category				
−3 to −2	74 (16.1)	54 (11.7)	128 (13.9)	.051
−2 to −1	385 (83.7)	408 (88.3)	793 (86)	
−1 to 0	1 (0.2)	0 (0)	1 (0.1)	
HAZ				
Mean (SD)	−1.52 (0.48)	−1.48 (0.41)	−1.50 (0.44)	.17
Range	−2.96 to −0.45	−3.00 to −1.00	−3.00 to −0.45	
BMI, kg/m^**2**^				
Mean (SD)	15.47 (2.29)	15.12 (2.55)	15.29 (2.43)	.03
Range	9.39-23.68	10.14-27.09	9.39-27.09	

a HAZ: height-for-age *z* scores.

### Baseline Nutrient Status of Study Participants

Tables4  5 summarize the baseline micronutrient status of the study participants. The findings indicate a considerable burden of micronutrient inadequacy in these participants. The most frequently observed inadequacy was vitamin D (97.5%), followed by folate (79.2%), zinc (66%), and vitamin A (34.3%; Table 6). An overview of the 24-hour macronutrient intake status is depicted in Table 7. The nutritional intake status was similar between both the study groups (Tables4  5).

**Table 4.** Summary of micronutrient status of the study participants at baseline (mITT^a^ population).

Parameters	Reference range^b^	Test group (n=445), mean (SD)	Control group (n=462), mean (SD)	Total (n=907), mean (SD)	*P* value
Serum vitamin A	26.00‐49.00 μg/dL	29.52 (6.30)	28.14 (5.41)	28.82 (5.89)	<.001
Serum vitamin B12	312.00‐1237.00 pg/mL	374.38 (87.31)	375.67 (97.51)	375.04 (92.59)	.83
Serum vitamin D	30.00‐100.00 ng/mL	17.48 (5.63)	17.89 (5.10)	17.69 (5.37)	.25
Serum vitamin E	0.30‐0.90 mg/dL	0.49 (0.14)	0.47 (0.16)	0.48 (0.15)	.046
Serum folate	5.00‐21.00 ng/mL	4.51 (0.73)	4.42 (0.71)	4.46 (0.72)	.06
Serum selenium	55.00‐134.00 μg/L	83.63 (12.61)	85.91 (12.24)	84.79 (12.47)	.01
Serum zinc	78.00‐105.00 μg/dL (aged 7‐9 y), 78.00‐118.00 μg/dL (females aged 10 y), 78.00‐98.00 μg/dL (males aged 10 y)	72.17 (19.80)	72.58 (19.98)	72.38 (19.88)	.76
Serum copper	51.00‐121.00 μg/dL	117.89 (15.92)	117.77 (16.08)	117.83 (15.99)	.91
Serum iron	50.00‐120.00 μg/dL	70.09 (29.77)	71.43 (29.14)	70.77 (29.44)	.49

a mITT: modified intention-to-treat.

b As per the Intervein Lab, Ahmedabad, India, a National Accreditation Board for Testing and Calibration Laboratories–accredited lab.

**Table 5.** Summary of micronutrient inadequacy status in the study participants at baseline (safety population).

Parameter	Reference range	Status of micronutrient inadequacy, n (%)				*P* value
		Abnormality	Test group (n=460)	Control group (n=462)	Total (n=922)	
Serum vitamin A	26.00‐49.00 μg/dL	Low	146 (31.7)	170 (36.8)	316 (34.3)	.11
Serum vitamin B12	312.00‐1237.00 pg/mL	Low	121 (26.3)	148 (32)	269 (29.2)	.06
Serum vitamin D	30.00‐100.00 ng/mL	Low	449 (97.6)	450 (97.4)	899 (97.5)	>.99
Serum vitamin E	0.30‐0.90 mg/dL	Low	32 (7.0)	53 (11.5)	85 (9.2)	.02
Serum folate	5.00‐21.00 ng/mL	Low	354 (77)	376 (81.4)	730 (79.2)	.10
Serum selenium	55.00‐134.00 μg/L	Low	1 (0.2)	0 (0)	1 (0.1)	.50
Serum zinc	78.00‐105.00 μg/dL (aged 7‐9 y), 78.00‐118.00 (females aged 10 y), 78.00‐98.00 (males aged 10 y)	Low	307 (66.7)	302 (65.4)	609 (66)	.68
Serum copper	51.00‐121.00 μg/dL	Low	0 (0)	1 (0.2)	1 (0.1)	>.99
Serum iron	50.00‐120.00 μg/dL	Low	133 (28.9)	121 (26.2)	254 (27.6)	.38

**Table 6.** Prevalence of micronutrient inadequacy in Indian school-age children (current study vs literature findings).

Micronutrients	Prevalence of micronutrient inadequacies		
Current study, %	Individual studies from literature, %	CNNS^a^ 2019 [9], %
Serum vitamin A	34.3	9.8‐43.9 [37,55,56]	22
Serum vitamin B12	29.2	7.4‐67.2 [37,57,58]	17
Serum vitamin D	97.5	14.2‐93.0 [37,56-62]	18
Serum vitamin E	9.2	No data found	Not reported
Serum folate	79.2	1.5‐99 [37,57,63,64]	28
Serum selenium	0.1	No data found	Not reported
Serum zinc	66	0.7‐57.1 [37,64,65]	17
Serum copper	0.1	No data found	Not reported
Serum iron (serum ferritin)	27.6	24.1‐54.5 [58,64]	17

a CNNS: Comprehensive National Nutrition Survey.

**Table 7.** Summary of macronutrient status of the study participants at baseline (mITT ^a^ population).

Parameters	RDA^b^	Test group (n=445)		Control group (n=462)		Total participants (n=907)	*P* value
Mean (SD)	% RDA	Mean (SD)	% RDA	Mean (SD)
Energy (kcal/day)	1690	1395.14 (305.78)	82.5	1406.08 (352.75)	83.2	1400.71 (330.40)	.62
Protein (g/day)	29.5	38.84 (11.88)	131.6	42.41 (13.39)	143.8	40.66 (12.79)	<.001
Carbohydrates (g/day)	N/A^c^	189.48 (50.75)	N/A	202.39 (55.08)	N/A	196.05 (53.36)	<.001
Fats (g/day)	30	47.52 (16.57)	158.4	40.03 (16.41)	133.4	43.71 (16.89)	<.001

a mITT: modified intention-to-treat.

b RDA: recommended dietary allowance.

c N/A: not applicable.

Since nutritional status is usually influenced by gut health, the study participants were also evaluated for their gut integrity through the lactulose:mannitol test and urinary neopterin test. A total of 111 (12%) participants demonstrated an abnormal lactulose:mannitol ratio (reference level: <0.035; test: n=66, 14.3%; control: n=45, 9.7%), indicating malabsorption in these participants (Table 8). On the other hand, the urinary neopterin test was normal for all the study participants across both groups (reference range: 0.10‐5.00 mmol/mol creatinine).

**Table 8.** Summary of baseline gut integrity/health using the lactulose:mannitol test and urinary neopterin test (mITT^a^ population).

Parameter	Test group	Control group	*P* value
**Lactulose mannitol test**			.002
Mean (SD)	0.0504 (0.19891)	0.0218 (0.01150)
Overall range min-max	0.000-2.200	0.000-0.132
**Urine neopterin test**			.27
Mean (SD)	0.419 (0.3967)	0.388 (0.4410)
Overall range min-max	0.10‐2.78	0.10‐4.22

a mITT: modified intention-to-treat.

## Discussion

### Principal Findings

This study revealed concerning facts about nutritional and growth parameters in the apparently healthy school-age children. The biochemical statuses of many micronutrients, namely vitamin A (34%), vitamin B12 (29%), vitamin D (97%), folate (79%), zinc (66%), and iron (28%) were observed to be inadequate in the children. In this study, we also assessed the gut health of the study participants. There is a paucity of prevalence data on this parameter in apparently healthy children, particularly those without diarrhea. We observed an abnormal lactulose:mannitol ratio in a substantial percentage (111/922, 12%) of the study population. This indicates compromised intestinal permeability, thus affecting their nutrient absorption.

This study found high compliance with the test product (99.99%), with a high retention rate of 98% (907/924). Maintaining similar influence and motivation among children and parents, particularly in an open-label individual RCT, could have been very challenging. However, this being a cluster/school-level intervention, peer influence could have helped improve compliance and adherence. Strategical measures used in the study to improve retention included weekly compliance monitoring, regular meetings and briefings with parents at the school, random home visits, and telephonic follow-up with nonresponders or during out-of-station travels.

Statistical implications of this study’s cluster RCT design necessitated inflating the sample size by 1.5 times the number needed for an individual RCT. Despite a relatively large sample size and a 9-month duration, the study was administratively more efficient because of the greater logistical convenience resulting from the operational ease of administration of the study intervention at the cluster level.

The magnitudes of nutritional inadequacies observed in this study were in line with most of the previously published literature (Table 8), reaffirming the presence of nutritional concerns in Indian school-age children [9,37,55-65]. However, when compared with CNNS estimates, the observed magnitudes of vitamin D, folate, and zinc deficiencies were relatively high. This may be due to higher cutoffs for serum vitamin D, folate, and zinc used in this study compared to the CNNS. Cutoffs in this study versus the CNNS are as follows: 30 ng/mL versus 12 ng/mL for vitamin D, 5 ng/mL versus 4 ng/mL for folate (equivalent to the cutoff of 151 ng/mL of red blood cell folate used in the CNNS), and 78 mcg/mL versus 65 mcg/mL for zinc. As a result, a greater number of children were included in the nutrient-deficient group in this study. Moreover, due to sedentary and indoor lifestyles, vitamin D deficiency is known to occur more commonly among children in urban settings [66,67], as in this study conducted with participants residing in the urban setting of Pune city.

This was one of the largest nutritional intervention trials conducted with a matched-pair cluster RCT design on Indian school-age children. Cluster trials are conceptually able to improve acceptability and adherence to an assigned intervention, but these qualities are rarely assessed and reported in cluster trial publications [68,69]. In a long nutritional intervention trial, consumption fatigue or boredom can set in, resulting in poor compliance and subsequent attrition from the study. This study recorded high compliance with the test product, with a high retention rate. Maintaining similar influence and motivation among children and parents, particularly in an open-label individual RCT, could have been very challenging. However, this being a cluster/school-level intervention, peer influence could have helped improve compliance and adherence. Besides, strategic measures used in the study including weekly compliance monitoring; regular meetings and briefings with parents at the school; random home visits; and telephonic follow-up with nonresponders or during out-of-station travels had an impact on retention.

### Limitations

The open-label design used in the study had great potential to pose a threat to the validity by introducing potential biases associated with such a design, but this was curtailed significantly by using specific and objective criteria for the diagnosing and recording of morbidities by physicians. Besides, results based on biochemistry were also objective by nature. Additionally, access to the treatment code was appropriately restricted during the process of blinded data review involving clinical research scientists and statisticians. The study involved the random allocation of clusters (ie, schools); however, participants’ selection within a school was not random due to the cluster RCT design. Prior knowledge of treatment could still have led to recruitment bias, which cluster RCTs are usually vulnerable to [70]. However, the study did not seem to have been impacted by this bias, as shown by the high screening to enrollment ratio, with a low proportion of participants refusing consent during screening in both the control group (16/482, 1.3%) and test group (6/476, 3.3%).

Although study groups were matched for overall socioeconomic status, the extent to which individual participants were matched was not ascertained. There was always a chance that potential socioeconomic status differences and associated factors (eg, access to health care, use of medications, hygiene, and sanitation practices) had the potential to confound the results. This was minimized by harmonizing the treatment of all morbidities by similarly trained study physicians in schools as a part of adverse event management. It was known that the use of medications could impact study outcomes, particularly ill days, school absenteeism, and illness severity. Thus, the medications (eg, antibiotics, antipyretics) were standardized across all study sites, and were thus eventually similar across both study groups. Since there were different study physicians at all 4 sites, an interobserver bias cannot be excluded. This was mitigated by making objective study end points and the physician’s discretion was used only while ruling out the allergic etiology of the illnesses. In addition, the training of the study physicians was harmonized with respect to quality, content, and duration.

Cluster randomization has statistical implications. The sample size was adjusted and planned to be increased 1.5 times compared to that needed for an individual RCT. In the primary analysis, the observed ICC, which measures the relatedness of clustered responses, was higher than anticipated, corresponding to a design effect more than 10 times higher than the protocol assumption. It is, however, comparable with estimates from the literature [71]. A higher number of clusters, with the same overall number of participants, could have increased statistical power. However, fewer clusters were used in the study due to practical reasons.

### Conclusions

The study findings highlight the extent of nutritional inadequacies in urban school-age children in India, reaffirming the need for nutritional strategies to optimize the nutritional status among these children.

This study suggested that a cluster RCT design can be effectively used in nutritional intervention trials. The design mitigates the ethical issues inherent in double-blind individual RCTs in children involving daily administration of an empty-calorie placebo. The strategies used to achieve high recruitment and retention rates are valuable for the conduct of future studies in a similar population.

## Supplementary material

10.2196/54189 Multimedia Appendix 1 Supplementary table

10.2196/54189 Checklist 1 CONSORT checklist 2025. CONSORT: Consolidated Standards of Reporting Trials.

## Abbreviations

AE: adverse event
ANCOVA: analysis of covariance
CNNS: Comprehensive National Nutrition Survey
DE: design effect
FMBF: fortified malt-based food
GI: gastrointestinal
HAZ: height-for-age *z* scores
Hb: hemoglobin
ICC: intracluster correlation coefficient
IDDS: individual dietary diversity score
ITT: intention-to-treat
LAR: legally acceptable representative
mITT: modified intention-to-treat
RCT: randomized controlled trial
SPWR: study physician weekly review
